# Protective Renal Effects of Atrial Natriuretic Peptide: Where Are We Now?

**DOI:** 10.3389/fphys.2021.680213

**Published:** 2021-05-28

**Authors:** Marcelo Roberto Choi, Belisario Enrique Fernández

**Affiliations:** ^1^Instituto Alberto C. Taquini de Investigaciones en Medicina Traslacional (IATIMET), CONICET - Universidad de Buenos Aires, Buenos Aires, Argentina; ^2^Departamento de Ciencias Biológicas, Facultad de Farmacia y Bioquímica, Cátedra de Anatomía e Histología, Universidad de Buenos Aires, Buenos Aires, Argentina; ^3^Instituto Universitario de Ciencias de la Salud, Fundación H.A. Barceló, Buenos Aires, Argentina

**Keywords:** atrial natriuretic peptide, nephroprotection, kidney, natriuresis, antioxidant

## Abstract

Atrial natriuretic peptide belongs to the family of natriuretic peptides, a system with natriuretic, diuretic, and vasodilator effects that opposes to renin-angiotensin system. In addition to its classic actions, atrial natriuretic peptide exerts a nephroprotective effect given its antioxidant and anti-inflammatory properties, turning it as a beneficial agent against acute and chronic kidney diseases. This minireview describes the most relevant aspects of atrial natriuretic peptide in the kidney, including its renal synthesis, physiological actions through specific receptors, the importance of its metabolism, and its potential use in different pathological scenarios.

## Introduction

The natriuretic peptide system involves the family of natriuretic peptides ANP, BNP, CNP, DNP, and urodilatin; the receptors NPR-A, NPR-B, and NPR-C; and the enzymes involved in their synthesis (furin, corin, and PCSK6) and degradation [neutral endopeptidase (NEP)] (Rukavina Mikusic et al., 2018b; Rubattu et al., 2019). Its main functions are to regulate hydro-saline homeostasis and blood pressure through renal actions combined with vascular effects. This system opposes the renin-angiotensin system (RAS), thus representing a protective mechanism against hypertension and their associated pathologies, including kidney damage (Rubattu et al., 2019). ANP was discovered in 1981 by de Bold et al., (1981) as a 28-amino acid peptide that is synthesized and stored in atrial and ventricular myocytes and released in response to various stimuli, such as stretching of the cardiac wall, endothelin, diverse cytokines, or adrenergic agents (Rukavina Mikusic et al., 2018b). Subsequently, it has been described that the kidney has all the biosynthetic machinery to produce ANP, its receptors, and the catabolic enzymes to degrade it (Wu et al., 2009; Choi et al., 2014). Given its actions in the kidney, it has been proposed that ANP could be involved in the pathogenesis of chronic kidney disease (CKD).

### Synthesis, Receptors, and Degradation of ANP

Corin is a type II transmembrane serine protease responsible for the cleavage of inactive pro-atrial natriuretic peptide (pro-ANP) into active ANP (Ricciardi et al., 2016). Synthesized as a zymogen, corin needs to be cleaved by a proprotein convertase subtilisin/kexin-6 (PCSK6, also called PACE4) in order to be activated (Chen et al., 2018). The kidney is the second organ with the highest amount of corin after the heart (Xue et al., 2020). Corin expression has been detected in the proximal tubule, the distal tubule (largely in the thick ascending medullary loop), and the collecting duct (especially in the internal medullary collector duct; Theilig and Wu, 2015). Several facts reveal the importance of corin or PCSK6 in the metabolic pathway of ANP and its effects on sodium homeostasis and blood pressure: (1) mutations in corin gene that affect its activation are associated with hypertension and preeclampsia (Cui et al., 2012; Dong et al., 2013), (2) in CKD patients, as well as proteinuric rats, it has been described reduced levels of corin in the kidney (Polzin et al., 2010; Fang et al., 2013), and (3) PCSK6 KO mice develop salt-sensitive hypertension and exhibit undetectable levels of corin and pro-auricular ANP activity (Chen et al., 2015).

To date, three types of natriuretic peptide receptors have been described: NPR-A, NPR-B, and NPR-C. ANP binds preferentially to NPR-A and NPR-C receptors (Rukavina Mikusic et al., 2018b). The NPR-A receptor is a transmembrane receptor encoded by the Npr1 gene, with its mRNA expressed in different organs, including the kidney. In the kidney, NPR-A is located at the renal vessels, podocytes, mesangial cells, proximal tubule, thin and thick ascending loop of Henle, collecting duct, and juxtaglomerular cells (Ogawa et al., 2012; Staffel et al., 2017). The guanylyl cyclase activity of NPR-A catalyzes the production of the second messenger cyclic guanosine monophosphate (cGMP), which triggers downstream activation of cGMP-dependent protein kinases (PKG) I and II, cyclic nucleotide-activated ion channels, and cyclic nucleotide phosphodiesterase (Beavo and Brunton, 2002). NPR-A displays a high affinity for ANP, BNP, and urodilatin, and its activation not only mediates vasodilator and natriuretic effects but also activates cellular mechanisms that regulate cell growth, apoptosis, proliferation, and inflammation (Rukavina Mikusic et al., 2018b). Several factors can regulate the expression of NPR-A, such as angiotensin II (Ang II), vitamin D, endothelin, endothelial NO synthase (eNOS), p38 MAPK, and osmotic stimuli. Also, the same ANP can negatively regulate its own Npr1 gene expression through cGMP response element-binding protein (Theilig and Wu, 2015). Regarding NPR-C, two subtypes with different molecular weights have been identified. The 77 kDa NPR-C lacks the guanylyl cyclase activity since it has a short intracellular domain, acting more as a clearance receptor through the internalization and degradation of natriuretic peptides (Theilig and Wu, 2015; Zhao and Pei, 2020). On the other hand, the 67 kDa NPR-C acts as an inhibitor of the adenylyl cyclase activity by coupling to an inhibitory Gi protein and activation of phospholipase C to exert multiple effects at the vascular, cardiac, metabolic, and bone (Rubattu et al., 2019). Recently, it has been demonstrated that both NPR-A and NPR-C mediate the renal effects of ANP by enhancing Ca2^+^/calmodulin-dependent NOS activity. NPR-A-induced NO production was shown to be cGMP dependent, while NPR-C-dependent NO release is partially mediated by Gi protein (Theilig and Wu, 2015).

ANP can be catabolized by two different pathways: (1) ANP binding to NPR-C receptor leads to ANP-NPR-C complex, which is internalized, and then, ANP is cleaved by lysosomal actions and (2) circulating ANP can be degraded by the NEP, neprilysin (Yandle et al., 1989; Nussenzveig et al., 1990). Neprilysin is a zinc-dependent metallopeptidase expressed in different tissues, including the kidney. This catabolic pathway takes relevance in scenarios with high levels of ANP, such as in heart failure (Potter, 2011). Although the main substrate for neprilysin is ANP, given its high complementary affinity, it also degrades other substrates, and thereby, its inhibition is not only associated with increased levels of ANP but also with other substances with vasodilator and natriuretic properties, as bradykinin, adrenomedullin, and angiotensin 1-7 (Volpe et al., 2019). In contrast, NEP inhibition can increase Ang II and endothelin-1 levels, blocking the beneficial effects of ANP through their vasoconstrictor and pro-fibrotic effects (Ferro et al., 1998). Recently, it has been reported that dipeptidyl peptidase-4 (DPP-4) would also degrade ANP, since inhibitors of DPP-4 exhibit antioxidant and anti-inflammatory effects, partially due to rising ANP levels and eNOS activity (Kamel et al., 2019).

### Renal Physiological Actions and Its Role as a Nephroprotector Hormone

**Figure 1:**
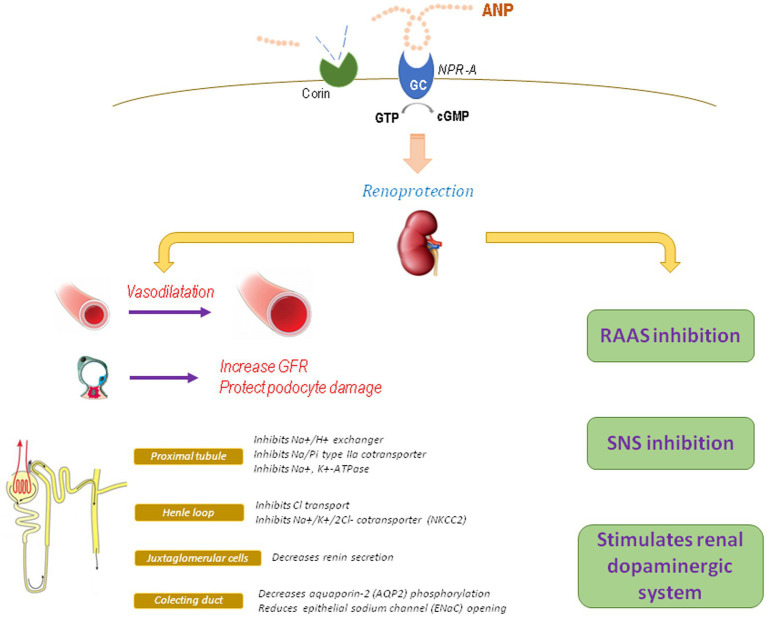
Physiological actions of ANP. GFR, glomerular filtration rate; RAAS, renin-angiotensin-aldosterone system; SNS, sympathetic nervous system.

In the kidney, ANP stimulates diuresis and natriuresis by different mechanisms (Figure 1): (1) At glomerular level: ANP vasodilates the afferent arteriole and contracts the efferent arteriole, thus increasing glomerular capillary pressure and therefore improving glomerular filtration rate (GFR) and fractional filtration (Dunn et al., 1986). This hemodynamic effect on the glomerulus is a consequence of ANP actions on the mesangial and smooth muscle cells of the renal arterioles. On podocytes, ANP exerts a protective effect, as it was demonstrated in mice with specific KO of NPR-A in podocytes and exposed to deoxycorticosterone acetate and high salt intake, where markedly increased levels of albuminuria and glomerular damage were observed related to a decrease in podocin, nephrin, and synaptopodin expression in the slit diaphragm and a greater influx of calcium ATP due to increased expression of canonical channel 6 (TRPC6; Ogawa et al., 2012; Staffel et al., 2017).

(2) At tubular level: ANP inhibits sodium reabsorption throughout the nephron (Choi et al., 2014). In the proximal tubule, ANP inhibits different Na^+^ transporters, as the Na^+^/H^+^ exchanger, Na/Pi type IIa cotransporter, and the Na^+^, K^+^-ATPase, and also counteracts angiotensin-stimulated sodium reabsorption (Theilig and Wu, 2015). Additionally, ANP can regulate other transporters as organic cation transporters (OCTs) and chloride channels (Darvish et al., 1995; Kouyoumdzian et al., 2016). In Henle’s thick ascending loop, ANP inhibits Cl^−^ transport and the activity and expression of the Na^+^/K^+^/2Cl^−^ cotransporter, reducing the ability to concentrate urine and increasing urine formation (Theilig and Wu, 2015). In the cortical collecting duct, ANP decreases aquaporin-2 phosphorylation, thus reducing vasopressin-dependent water permeability (Dillingham and Anderson, 1986; Klokkers et al., 2009). In the medullary collecting duct, acting on main cells, ANP reduces epithelial sodium channel (ENaC) opening as well as other sodium channels as cyclic nucleotide-regulated cation channels (CNG; Light et al., 1990, Guo et al., 2013). The ENaC channel is a key target for ANP, since corin knockout mice display an increase in the β subunit of medullary ENaC with enhanced reabsorption of sodium and water and development of salt-sensitive hypertension (Polzin et al., 2010; Wang et al., 2012).

(3) At hormonal level: ANP has antagonic effects on the RAS, which contributes to its nephroprotective actions, since ANP decreases renin secretion from juxtaglomerular cells, reduces Ang II and vasopressin effects, and blocks aldosterone synthesis and release (Kurtz et al., 1986; Brenner et al., 1990; Volpe et al., 2019). Aldosterone is a determining factor for CKD progression. As a physiological antagonist, ANP can block aldosterone-induced MAPK phosphorylation in podocytes and also avoid aldosterone-induced nuclear translocation of the mineralocorticoid receptor *via* PKG I (Kato et al., 2017). Additionally, hypertension and glomerular injury (mesangial expansion, segmental sclerosis, severe podocyte injury, and increased oxidative stress) induced by chronic administration of aldosterone and high salt diet are deeply impaired and accelerated in mice lacking of NPR-A receptor, even when blood pressure is controlled with hydralazine, suggesting that these effects are independent of hypertension (Ogawa et al., 2012). All these alterations can be reduced by the AT1 receptor blocker olmesartan or with the antioxidant tempol, suggesting that the nephroprotective properties of ANP are related to its antagonic action against the RAS as well as its antioxidant effects (Baradaran et al., 2014; Rukavina Mikusic et al., 2014). It has also been reported that ANP is capable to regulate another local natriuretic system, the renal dopaminergic system. In this way, the diuretic and natriuretic effects of ANP are partially exerted by favoring the recruitment of D1R receptors and by enhancing the inhibitory effect of dopamine on Na^+^/H^+^ exchanger in the proximal tubules (Choi et al., 2014). Furthermore, ANP stimulates tubular dopamine uptake *via* NPR-A, cGMP, and PKG, increases the activity of dopa decarboxylase, and reduces the activity of COMT, thus favoring the tubular bioavailability of dopamine (Fernández et al., 2005; Correa et al., 2007, 2008). These effects were reproduced in an experimental *in vivo* model, in which the infusion of ANP at low doses increased the urinary excretion of dopamine by stimulating the activity of OCTs with subsequent over-inhibition of the Na^+^, K^+^-ATPase (Kouyoumdzian et al., 2016). Additionally, Ang II opposes to ANP by downregulating the renal dopaminergic system (Rukavina Mikusic et al., 2018a; Kouyoumdzian et al., 2021). This synergic interaction between ANP and renal dopamine not only favors the maintenance of hydro-electrolyte balance, blood pressure, and a stable redox state, but also its alteration would be implicated in the pathophysiology of arterial hypertension and inflammatory renal damage (Armando et al., 2011; Choi et al., 2014).

**Figure 2:**
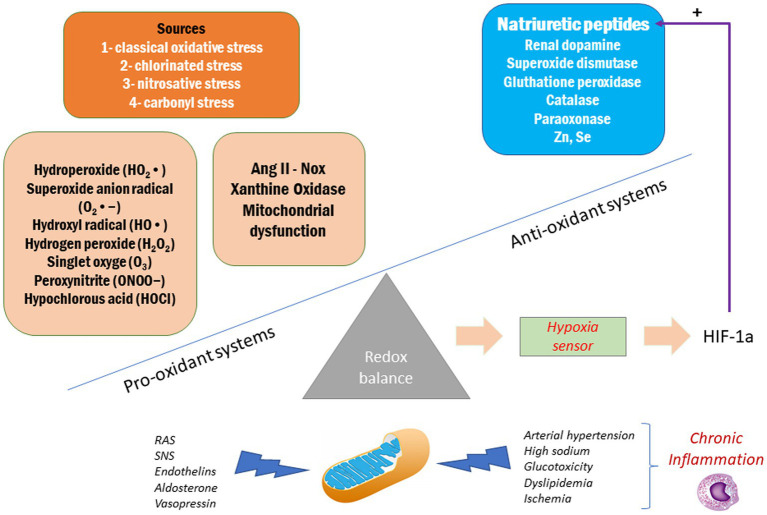
Oxidative stress in the progression of chronic kidney disease.

In addition to its hemodynamic and renal effects, it has been described that ANP exhibits antioxidant and anti-inflammatory properties that justify its nephroprotective effects (Rukavina Mikusic et al., 2014). Oxidative stress and inflammation are determining factors for the development of kidney damage (Harrison et al., 2011; Figure 2). It has been shown that ANP is capable of attenuate ROS levels in different models of kidney injury such as animals fed with chronic high sodium diet, in which the hypoxia generated increases the levels of HIF-1α, an important stimulus for ANP synthesis and release (Chun et al., 2003; Della Penna et al., 2014). In addition, it has been demonstrated that ANP exhibits anti-inflammatory effects by inhibiting the activation of the nuclear factor NF-kB (by inducing the expression of the inhibitor IκB of NF-κB), iNOS, RANTES, cyclooxygenase-2, and TNF-α, thus decreasing the production of peroxynitrites, cytokines, and chemokines (Kiemer and Vollmar, 1998; Kiemer et al., 2002a,b). Additionally, ANP exerts an indirect effect by facilitating the action of the renal dopaminergic system, which also exhibits an antioxidant effect by inhibiting NADPH oxidase and stimulating the antioxidant enzymes superoxide dismutase, glutathione peroxidase, glutamyl cysteine transferase, Parkinson’s protein 7 (PARK7 or DJ-1), paraoxonase 2 and heme oxygenases 1 and 2 (George et al., 2012; Yang et al., 2012; Rukavina Mikusic et al., 2014).

### ANP as a Renal Therapeutic Agent

Several experimental and clinical studies have demonstrated the benefit of ANP administration in the prevention or reversion of renal damage. Given its vasodilator and renal hemodynamic effects, the ANP analog, anaritide, has been shown to improve diuresis in patients with oliguric acute renal failure and reduce the need for dialysis (Allgren et al., 1997). In the renal ischemia-reperfusion injury model, the infusion of ANP (0.2 mg/kg/min) prevented metabolic acidosis, increased plasma creatinine and lactate, reduced tubular injury, increased activity of eNOS, and attenuated the expression of TNF-a in the kidney (Chujo et al., 2010; Tulafu et al., 2014). On the other hand, the acute kidney injury developed during surgery is associated with a greater risk of adverse events and death; therefore, strategies that protect the kidney are of interest to prevent or reduce them. In this context, it has been reported that the administration of ANP, especially in low doses, exerts beneficial and protective effects against acute kidney injury after cardiac surgery by preserving renal function, increasing intraoperative diuresis, and improving the degree of acute renal failure (Mitaka et al., 2011; Moriyama et al., 2017). In addition, the infusion of ANP or its analog, carperitide, in CKD patients undergoing cardiac surgery reduces postoperative serum creatinine concentration both in the acute postoperative stage and even up to 1 year after surgery with a lower rate of postoperative cardiac events and need of dialysis (Sezai et al., 2011; Mori et al., 2014). In contrast, a prospective, multicenter, randomized, double-blind, and placebo-controlled clinical study, carried out in 77 patients with acute kidney injury associated with cardiac surgery, demonstrated that a low-dose ANP infusion of 0.02 μg/kg/min significantly increased urine output but failed to improve kidney function. The authors postulate that this lack of response observed with ANP could be due to the fact that in the pathophysiological process of acute injury associated with cardiac surgery, renal blood flow decreases due to several mechanisms such as ischemia and reperfusion injury, neurohormonal activation, pro-inflammatory mediators and vasoconstrictor agents, oxidative stress, and the presence of exogenous and endogenous toxins (Mitaka et al., 2017).

The renal benefits of ANP have also been demonstrated in the initial oliguric phase of endotoxemia and in renal failure induced by nephrotoxic agents. In this way, the addition of carperitide (a recombinant ANP) at a dose of 1.8 μg/kg/h to fluid resuscitation therapy significantly improved both the glomerular filtration and the tubular flow rates in rats exposed to lipopolysaccharides (Kitamura et al., 2018). Additionally, subcutaneous infusion of ANP in rats at 1.5 μg/kg/min was shown to significantly reduce the cisplatin-induced increase in creatinine and urea nitrogen, the urine albumin/creatinine ratio, tubular necrosis, and renal expression of inflammatory markers, such as IL-1β, IL-6, intercellular adhesion molecule 1, and monocyte chemoattractant protein 1 mRNA (Nojiri et al., 2015).

### The Imbalance Between ANP and Ang II as a Potential Nephroprotective Strategy in Chronic Kidney Disease

Chronic kidney disease is associated with an imbalance of vasoactive substances in the kidney, with an increase in vasoconstrictor agents, such as Ang II and endothelin-1, and a reduction in vasodilator agents, such as nitric oxide, bradykinin, and ANP, which causes renal hemodynamics alterations and intraglomerular capillary hypertension (Benigni et al., 2004). Considering the beneficial effects of ANP in the kidney and the deleterious effects of the RAS, the use of a combined inhibition that favors the protective actions of ANP and blocks the harmful effects of Ang II represents an interesting approach to evaluate. In this way, the dual inhibition of the angiotensin receptor AT1 and neprilysin (called ARNI) is a novel therapy that combines a neprilysin inhibitor (sacubitril) that enhances the action of ANP, and a selective antagonist of the AT1 receptor (valsartan) that counteracts the increase in Ang II induced by sacubitril while avoiding the incidence of cough and angioedema caused by ACE inhibitors (Uijl et al., 2020). ARNIs are currently indicated for patients with heart failure (HF) with reduced ejection fraction, where they have shown benefits in terms of morbidity and mortality (McMurray et al., 2014). Experimentally, in rats undergoing 5/6 nephrectomy, chronic use of the dual inhibitor sacubitril-valsartan for 8 weeks was associated with cardiorenal benefit by reducing cardiac hypertrophy, aortic fibrosis, and improvement of renal function (Suematsu et al., 2018). Another study demonstrated that treatment with ARNI was associated with increased ANP levels and significant protection against kidney damage in SHRSP rats compared to valsartan alone (Rubattu et al., 2018). Other studies that used an experimental model of angiotensin II-dependent hypertensive diabetic rats [TGR (mREN2) 27 rats] demonstrated that chronic treatment with sacubitril/valsartan was associated with increased in urinary ANP levels, reduced albuminuria, and less development of segmental glomerulosclerosis compared to valsartan monotherapy (Roksnoer, 2016; Uijl et al., 2020). This renoprotective effect of ANP would be independent of its antihypertensive efficacy and could be related to a reduction in renal inflammation. The benefits of inhibiting neprilysin are not limited only to glomerular protection but also extend to the tubulointerstitial fibrosis. It was demonstrated in renomedullary interstitial cells from neprilysin knockout mice exposed to a hyperglycemic environment that ANP reduced cell proliferation and extracellular matrix synthesis induced by Ang II, being this effect more pronounced in the presence of a selective antagonist of the AT1 receptor (Maric et al., 2006). Finally, a recent systematic review and meta-analysis that included 10 randomized controlled trials (*n* = 16,456 patients) evaluated the renal outcome of ARNI against RAS inhibitors (ACE inhibitors or AT1 receptor blockers) in patients with or without HF (Spannella et al., 2020). Compared with RAS inhibitors alone, ARNI treatment resulted in a lower risk of renal dysfunction, especially with a strong association in older patients or HF patients with preserved ejection fraction. However, no significant association was found in patients without HF (Spannella et al., 2020).

Although, in experimental models of kidney damage, beneficial and superior effects of ARNI were observed compared to RAS inhibitors, the real impact of these agents in CKD patients is still unknown. In this sense, the United Kingdom Heart and Renal Protection-III trial was designed to evaluate if ARNI therapy can slow the rate of kidney decline better than current standard treatment with RAS inhibitor irbesartan in patient with CKD without HF (Haynes et al., 2018). This double-blind, randomized trial included 414 patients with moderate to severe CKD (estimated GFR 20 to 60 ml/min/1.73 m^2^) who were randomly assigned to sacubitril/valsartan 97/103 mg twice daily (*n* = 207) or irbesartan 300 mg once daily (*n* = 207). There was no difference in the primary outcome (GFR at 12 months) between both treatment arms urinary neither in the albumin:creatinine ratio. There was only a decrease in systolic and diastolic blood pressure and some cardiac biomarkers (troponin I and NT-proBNP) in the ARNI group against irbesartan, suggesting a possible role in the cardiovascular risk reduction in advanced CKD patients (Haynes et al., 2018). Therefore, conversely to what was reported experimentally, this clinical study indicates that ARNI therapy in patient with moderate to severe CKD has similar effects on kidney function and albuminuria than current standard treatment with RAS inhibitors. However, it is necessary to emphasize that this lack of response could be due to some limitations of this study, like short time of study, number of patients, and type and stage of CKD. Therefore, the evidence on the clinical benefits of ARNI in CKD regardless the presence of HF is still scarce, but enough to demonstrate its safety at the renal level.

## Conclusion

The expression of all the components of this natriuretic system in the kidney is relevant to maintain renal function and to exert a nephroprotective effect, and its alteration can predispose to kidney damage with increased levels of pro-inflammatory cytokines and oxidative stress, albuminuria, renal tubular injury, and interstitial fibrosis. Although most of the evidence supports a beneficial protecting renal effect of ANP or its analogs, some clinical trials reported no benefit in terms of kidney function improvement during cardiovascular surgery. Since ANP also antagonizes the renin-angiotensin-aldosterone system, its potential benefit could be extent to those situations in which exist an imbalance in favor of Ang II and in detriment of ANP such as CKD. Given the fact that CKD is a constantly growing entity and can progress over time to a greater renal and cardiovascular deterioration even under renoprotection with RAS inhibitors, the search for new treatments to improve renal protection is imperative. The beneficial effects of pharmacological use of dual angiotensin receptor AT1-neprilysin inhibitors are currently limited to HF patients, raising the urgent need to investigate the renal outcome of ARNI therapy outside the HF setting, such as type 2 diabetes mellitus and CKD, scenarios in which the evidence is still scarce and of low quality.

## Author Contributions

MC and BF wrote, designed, and revised the manuscript. All authors contributed to the article and approved the submitted version.

### Conflict of Interest

The authors declare that the research was conducted in the absence of any commercial or financial relationships that could be construed as a potential conflict of interest.
